# The Manifestation of Congenital Hypoplasia of the Depressor Anguli Oris: A Case Report of Asymmetric Crying Facies

**DOI:** 10.7759/cureus.78352

**Published:** 2025-02-01

**Authors:** Abhimanyu Vasudeva, Ruchika Agrawal, Anchala Bhardwaj, Swati Dwivedi, Vivek Mishra, Vikram Vardhan

**Affiliations:** 1 Physical Medicine and Rehabilitation, All India Institute of Medical Sciences, Gorakhpur, IND; 2 Otolaryngology, All India Institute of Medical Sciences, Gorakhpur, IND; 3 Pediatrics, All India Institute of Medical Sciences, Gorakhpur, IND; 4 General Practice, Avadh Diabetes and Heart Care Centre, Rudauli, IND; 5 Anatomy, All India Institute of Medical Sciences, Gorakhpur, IND; 6 Anaesthesiology, Pain Medicine and Critical Care, All India Institute of Medical Sciences, Gorakhpur, IND

**Keywords:** counseling, crying, facial asymmetry, facial muscles, infant

## Abstract

Congenital hypoplasia of the depressor anguli oris muscle (CHDAOM) is a relatively uncommon condition that causes asymmetric crying facies (ACF) in newborns. Although it primarily presents as a cosmetic issue, it could have implications in clinical practice. We report a case involving a seven-month-old girl who presented with asymmetric crying and other related clinical features. The patient exhibited lateral deviation of the mouth angle during smiling and crying but showed no additional clinical abnormalities. There were no signs of generalized facial muscle weakness or paralysis. The diagnosis of CHDAOM was confirmed, based on left-sided mouth deviation during smiling and crying, with no systemic manifestations. The parents reported the presence of deviation since birth. The asymmetry was clearly visible upon clinical examination. The clinical signs included the observed facial asymmetry, with no associated abnormal findings in other areas. The patient’s parents were counseled on the benign nature of the condition to alleviate any concerns. The report also includes a discussion of the management approach and addresses embryological considerations related to CHDAOM.

## Introduction

Neonatal asymmetric crying facies (ACF) is a unilateral facial asymmetry affecting approximately one in 160 live births. This condition can be caused by nerve compression during birth, which typically resolves on its own, or by developmental abnormalities. When developmental issues, such as congenital hypoplasia of the depressor anguli oris muscle (CHDAOM), are identified, a thorough evaluation is essential. These developmental abnormalities may be associated with other congenital anomalies, and persistent facial asymmetry may necessitate surgical intervention to enhance cosmetic appearance and functional outcomes as the child matures into adulthood [1]. A case report detailing the management of congenital unilateral hypoplasia of the depressor anguli oris (DAO) muscle in an adult has suggested that using bidirectional fascial grafts, applied both horizontally and vertically, could be a successful treatment strategy for adults with this condition [2].

The DAO muscle starts forming between the third and eighth weeks of embryonic development. During this period, the mesoderm in the second branchial arch thickens and develops into five laminae on each side of the face. These laminae give rise to various facial muscles, including the DAO. Specifically, the mandibular laminae are responsible for the development of this muscle, along with others such as the depressor labii inferioris, mentalis, risorius, buccinator, and levator anguli oris. Thus, the DAO originates from the mesodermal tissue associated with the mandibular region, playing a key role in shaping the facial musculature [3]. The DAO is a facial muscle situated in the outermost layer of the perioral region. It originates from the oblique line of the mandible and tapers as it extends, eventually converging with the modiolus. This muscle plays a key role in lowering the corner of the mouth [4]. The underlying reason for the hypoplasia of the DAO muscle is not yet fully understood [5]. Its diagnosis is primarily based on clinical assessment [6]. 

In cases of CHDAOM where surgery may be considered, it is crucial to have a detailed understanding of the facial artery’s branching patterns and its relationship to key surgical landmarks. Observations of gender-related differences, along with the asymmetrical distribution of these parameters, indicate that such variations must be accounted for during surgical planning. Moreover, the presence of various types of facial arteries, their potential absence, and other anatomical discrepancies should all be considered in the preoperative evaluation to ensure optimal surgical outcomes [7].

## Case presentation

A seven-month-old female was evaluated for a history of left-sided mouth deviation observed during crying since birth. The caregivers noted that the condition had not worsened over time and reported no family history of comparable issues. The child had achieved all anticipated developmental milestones and had not previously had any medical treatments or surgeries related to facial asymmetry. The mother's antenatal period had been uneventful, with no exposure to known teratogens, and there was no family history of this condition. The child, who was born at term with no interventions, was the firstborn to a 22-year-old mother.

**Figure 1:**
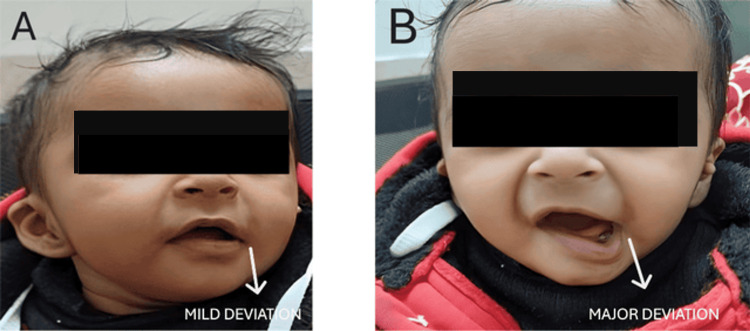
Images of the patient A: minor deviation; B: major deviation

Her birth weight was 2.6 kg, and she had cried immediately after birth. Clinical evaluation showed normal facial features and cranial nerve function, with no evidence of neurological abnormalities. The caregivers were mainly worried about the child’s social adaptability due to the facial asymmetry and potential underlying pathology. Physical examination revealed thinning of the lower lip on the affected side and deviation of the left corner of the mouth downward and laterally during crying, while the right corner remained stable (Figure 1).

The clinical examination revealed no additional anomalies, including craniofacial, skeletal, genitourinary, or cardiac abnormalities. The clinical presentation supported a diagnosis of CHDAOM, and the condition was explained to the parents as benign and non-threatening.

## Discussion

In CHDAOM, the mouth angle and mandible on the unaffected side are drawn downward due to the unopposed action of the normal DAO muscle, whereas there is no corresponding movement on the side affected by hypoplasia [8]. ACF can occur in isolation or may be linked to a range of anomalies affecting the cardiovascular, musculoskeletal, respiratory, gastrointestinal, central nervous, or genitourinary systems [9]. While the hypoplasia of the DAO muscle may occur as an isolated condition, its identification warrants a thorough search for potential coexisting anomalies [10].

Neonatal asymmetric crying facies, congenital facial paralysis, and developmental facial paralysis can all lead to noticeable facial deformities in newborns. An accurate diagnosis and thorough awareness of these conditions are crucial, as is understanding the stigma faced by parents. An article has sought to improve understanding, facilitate early detection, and support effective screening and counseling for affected families [11]. Having difficulty expressing emotions in a clear manner can create concerns for both the child and their parents [12]. An insightful and comprehensive article has described scanning techniques using high-resolution ultrasound for precise imaging of the facial muscles, facilitating the identification of asymmetries and enabling the evaluation of areas that may require intervention [13]. 

Urgent treatment may not be required for neonates with ACF who present with isolated facial anomalies, particularly when the cosmetic concerns are minimal and do not significantly affect the child [14]. Ultimately, it is important to distinguish this condition from other possible causes of facial asymmetry at birth, including fetal positioning or pressure on the stylomastoid foramen during labor, as both can contribute to facial paralysis and must be carefully considered during diagnosis [15]. In instances where surgical intervention is deemed necessary, it is crucial to account for the observed differences between sexes in facial and nasal dimensions, as well as facial indices. These variations are key to tailoring surgical strategies to ensure that they are appropriately adapted to the unique anatomical characteristics of each patient [16].

## Conclusions

This report discussing a case of CHDAOM underscores the significance of a comprehensive, multidisciplinary approach to both its diagnosis and management, particularly when engaging with parents who may have varying levels of understanding. Although the condition is benign and typically requires no intervention, the parents in our case, due to limited formal education, took several months to fully accept the diagnosis before reaching out to our team. This highlights the critical need for clear, empathetic, and patient-centered communication, especially when addressing rare conditions that may initially seem alarming. While surgical intervention is usually unnecessary, the report emphasizes the importance of continuous support and reassurance for families. A thorough history and clinical examination are essential in ensuring an accurate diagnosis and appropriate management. Ultimately, this report reinforces the value of a holistic approach to care that combines medical expertise with effective patient education.
